# Expanding the application of haplotype-based genomic predictions to the wild: A case of antibody response against *Teladorsagia circumcincta* in Soay sheep

**DOI:** 10.1186/s12864-023-09407-0

**Published:** 2023-06-17

**Authors:** Seyed Milad Vahedi, Siavash Salek Ardetani, Luiz F. Brito, Karim Karimi, Kian Pahlavan Afshari, Mohammad Hossein Banabazi

**Affiliations:** 1 Department of Animal Science and Aquaculture, Dalhousie University, Truro, NS B2N5E3 Canada; 2 Department of Animal Science, University of Zanjan, Zanjan, 4537138791 Iran; 3 Department of Animal Sciences, Purdue University, West Lafayette, IN 47907 USA; 4 Molecular Diagnostics Program, Verspeeten Clinical Genome Centre, London Health Sciences Centre, London, ON N6A 5W9 Canada; 5 Department of Animal Sciences, Islamic Azad University, Varamin, Varamin-Pishva Branch3381774895 Iran; 6 Department of Animal Breeding and Genetics (HGEN), Centre for Veterinary Medicine and Animal Science (VHC), Swedish University of Agricultural Sciences (SLU), 75007 Uppsala, Sweden; 7 Department of Biotechnology, Animal Science Research Institute of IRAN (ASRI), Agricultural Research, Education & Extension Organization (AREEO), Karaj, 3146618361 Iran

**Keywords:** Genomic prediction, Haplotype-based models, Pseudo-SNP, Individual SNP models, GBLUP, Bayesian, Soay sheep

## Abstract

**Background:**

Genomic prediction of breeding values (GP) has been adopted in evolutionary genomic studies to uncover microevolutionary processes of wild populations or improve captive breeding strategies. While recent evolutionary studies applied GP with individual single nucleotide polymorphism (SNP), haplotype-based GP could outperform individual SNP predictions through better capturing the linkage disequilibrium (LD) between the SNP and quantitative trait loci (QTL). This study aimed to evaluate the accuracy and bias of haplotype-based GP of immunoglobulin (Ig) A (IgA), IgE, and IgG against *Teladorsagia circumcincta* in lambs of an unmanaged sheep population (Soay breed) based on Genomic Best Linear Unbiased Prediction (GBLUP) and five Bayesian [BayesA, BayesB, BayesCπ, Bayesian Lasso (BayesL), and BayesR] methods.

**Results:**

The accuracy and bias of GPs using SNP, haplotypic pseudo-SNP from blocks with different LD thresholds (0.15, 0.2, 0.3, 0.4, 0.5, 0.6, 0.7, 0.8, 0.9, and 1.00), or the combinations of pseudo-SNPs and non-LD clustered SNPs were obtained. Across methods and marker sets, higher ranges of genomic estimated breeding values (GEBV) accuracies were observed for IgA (0.20 to 0.49), followed by IgE (0.08 to 0.20) and IgG (0.05 to 0.14). Considering the methods evaluated, up to 8% gains in GP accuracy of IgG were achieved using pseudo-SNPs compared to SNPs. Up to 3% gain in GP accuracy for IgA was also obtained using the combinations of the pseudo-SNPs with non-clustered SNPs in comparison to fitting individual SNP. No improvement in GP accuracy of IgE was observed using haplotypic pseudo-SNPs or their combination with non-clustered SNPs compared to individual SNP. Bayesian methods outperformed GBLUP for all traits. Most scenarios yielded lower accuracies for all traits with an increased LD threshold. GP models using haplotypic pseudo-SNPs predicted less-biased GEBVs mainly for IgG. For this trait, lower bias was observed with higher LD thresholds, whereas no distinct trend was observed for other traits with changes in LD.

**Conclusions:**

Haplotype information improves GP performance of anti-helminthic antibody traits of IgA and IgG compared to fitting individual SNP. The observed gains in the predictive performances indicate that haplotype-based methods could benefit GP of some traits in wild animal populations.

**Supplementary Information:**

The online version contains supplementary material available at 10.1186/s12864-023-09407-0.

## Background

Genomic prediction of breeding values (GP) was described over 20 years ago with the primary goal of accurately identifying the breeding candidates with the highest genetic merit using genome-wide single nucleotide polymorphism (SNP) markers [1]. The development of GP models and the affordable cost of genotyping have revolutionized animal breeding by increasing selection accuracies, shortening the generation interval, and increasing genetic progress of economically important traits [2, 3]. GP has also received considerable attention in other fields with similar practical needs. Genomic selection has been implemented in plant breeding programs for genetic improvement of quantitative traits [4, 5]. Moreover, GP has been applied to human genetics for identifying patient risk for particular diseases, referred to as “polygenic risk score” or selecting the best treatment option based on the individual’s genotype [6, 7]. Despite the application of GP in livestock, plant, and human genetics, a limited number of studies have applied this method in wild or unmanaged animal populations [8–10].

The choice of the statistical model to be used for GP is a critical step in the success of genomic analyses. Statistical models commonly used for GP can be classified into two categories: (i) linear parametric methods referred to as “Best Linear Unbiased Prediction (BLUP) methods”, such as genomic BLUP or GBLUP [11] and Single-step GBLUP [12], and (ii) non-linear parametric methods, such as BayesA [1], BayesB [1], BayesCπ [13], Bayesian Lasso (BayesL) [14], and BayesR [15]. These methods mainly differ in the assumptions used for the prior distribution of genetic effects. In GBLUP, a normal prior distribution is assumed for the marker effects, which means that a large number of quantitative trait loci (QTL) influence the trait, with most markers exhibiting a small effect [1]. The two models of BayesA and BayesB, described by Meuwissen et al. [1], assume SNP-specific variances; BayesA fits all SNPs, while BayesB fits approximately 1-π proportion of SNPs, where π is the percentage of markers which have no influence on the trait (zero effect). Therefore, when π = 0, BayesB is equivalent to BayesA. BayesCπ is similar to BayesB but treats π as an unknown parameter with a uniform (0, 1) prior distribution, and it assumes all SNP effects have a common variance [13]. The BayesL method assumes that the variance of the SNP marker effects follows a double exponential or Laplace distribution [16]. BayesR provides high flexibility by using a mixture of normal distributions as the prior for SNP effects [15]. In this method, four classes of SNP effect size (null, small, medium, and large) can be defined, for instance, and SNP effects would be modeled using a four-component normal mixture model [15]. In general, Bayesian approaches tend to be more accurate than GBLUP when the number of QTL explaining the trait variance is small [17].

Practical applications of GP have focused on single-SNP models fitting individual SNP as a locus in the mixed models without any information about the marker location. Instead, haplotype models have the potential to include structural genomic information to improve the accuracy of genomic evaluation [18–20]. Haplotypes are more informative than SNPs in describing recent identical-by-descent (IBD) relationships, and they may also be more effective in capturing linkage disequilibrium (LD) with multiallelic QTL than individual SNP, which are often biallelic [19]. In practice, the performance of GP based on haplotypes varies across traits and species, ranging from negligible to substantial increases in accuracy compared to SNP-based models [19, 21–24]. Three methods have been applied to define haploblocks, including (i) a fixed number of SNPs per haplotype block [25], (ii) fixed block length [26], and (iii) LD blocks [27]. The latter method is an efficient method that can decrease the number of explanatory variables without losing the information provided by the SNP markers [28]. By setting a minimum LD between SNP markers, they can be grouped into haploblocks that do not have a fixed length or a fixed number of SNPs. Due to the presence of relatively strong LD among particular markers, the number of variants per haploblock is reduced considerably compared to when haploblocks are defined by a fixed number of close SNPs [27]. Haplotypic information could be then integrated as pseudo-SNPs into BLUP [21, 22] or Bayesian [26] GP models, or based on a recent method applied by Araujo et al. [23], the pseudo-SNPs can contribute to a genomic relationship matrix (GRM) construction in combination with non-LD clustered SNPs, i.e., those located out of haploblocks.

Recently, GP methods have been adopted by researchers interested in quantitative evolution of wild animals [8, 9]. With the rise of wildlife infectious diseases, e.g., sea-star wasting disease [29], bats’ white-nose syndrome [30], chytrid fungus in amphibians [31], and the emergence of zoonotic infections such as SARS-CoV-2 in captive [32, 33] and unmanaged populations [34], GP models could be used to improve captive breeding and conservation strategies to select resistant individuals against pathogens. Moreover, GP models can be used in wild populations to investigate the microevolutionary trends of traits, and more accurate models can better demonstrate these changes. Then, we can better understand the “cryptic microevolution” process, which refers to traits being heritable and under directional selection, but they do not constantly evolve in response to that selection in the expected way [35]. Thus, it is of interest to know the accuracy of different GP models in prediction of the individuals’ genomic merit for different traits in wild populations. Additionally, it is unclear if the GP of immune response traits in wild populations would potentially benefit from haplotypic information.

The goal of the present study was to investigate the GP accuracy of antibody response against a gastrointestinal strongyle nematode, *Teladorsagia circumcincta*, in Soay sheep lambs, as an example to explore the performance of haplotype-based GP models for their potential future applications in captive breeding or conservation strategies. Therefore, three different analyses were performed: (i) SNP markers were fitted, (ii) haplotypes constructed based on different LD threshold were fitted as pseudo-SNPs, and (iii) the pseudo-SNPs combined with the non-LD clustered SNPs were fitted. The accuracy and bias of GP from GBLUP and five Bayesian approaches, including BayesA, BayesB, BayesCπ, BayesL, and BayesR, were then compared. This study used a publicly-available dataset from a 25-year study that quantified antibody levels in unmanaged Soay breed lambs [36].

## Results

The descriptive statistics of the phenotypic records and adjusted phenotypes are presented in Table 1. The average ± standard error (SE) of inter-marker distance was 68.50 ± 2.19 Kb, and the minimum and maximum distances between SNPs were 3.17 Kb and 423.72 Kb, respectively.

**Table 1:** Total number (N), minimum (Min), maximum (Max), mean, standard deviation (SD) of phenotypes and adjusted phenotypes, and number of individuals in the training^a^ and testing^b^ sets

Trait	N	Min	Max	Mean	SD	Training (N)^a^	Testing (N)^b^
IgA	2,034	0	2.73	0.74	0.50	1,848	186
IgA_adj^c^		-0.84	2.02	0	0.50		
IgE	2,034	0	1.08	0.09	0.12	1,848	186
IgE_adj^a^		-0.14	1.00	0	0.12		
IgG	2,034	0	1.60	0.24	0.19	1,848	186
IgG_adj^a^		-0.32	1.36	0	0.19		

^c^IgA_adj = adjusted phenotype of IgA; IgE_adj = adjusted phenotype of IgE; IgG_adj = adjusted phenotype of Ig

Haploblock construction was performed based on ten thresholds of LD (measured as r^2^) ranging from 0.15 to 1.00 (Table 2). As the LD threshold increased, the number of haploblocks and pseudo-SNPs decreased, ranging from 1,432 to 8,442 and 2,897 to 28,265, respectively. With an increase in ${r}^{2}$, the average number of SNPs per block decreased, ranging from 2.10 to 2.22 SNPs. Moreover, with stricter LD thresholds, the total number of SNPs applied to haploblocks and the total length of the autosome covered by the haplotypes decreased from 18,705 to 2,991 and 249.7 Mb to 32.2 Mb, respectively (Table 2).

**Table 2:** Statistics of haploblocks defined based on linkage disequilibrium (LD) levels of ${\mathbf{r}}^{2}$

	$${\mathbf{r}}^{2}$$									
	**0.15**	**0.20**	**0.30**	**0.40**	**0.50**	**0.60**	**0.70**	**0.80**	**0.90**	**1.00**
**Total number of pseudo-SNP**	28,265	26,673	22,971	19,562	16,384	13,354	10,663	8,031	5,816	2,897
**Number of blocks**	8,442	7,976	7,021	6,110	5,248	4,399	3,654	2,906	2,281	1,432
**Min SNPs per block**	2	2	2	2	2	2	2	2	2	2
**Max SNPs per block**	6	6	6	6	6	6	6	6	6	5
**Average number of SNPs per block**	2.22	2.19	2.17	2.16	2.15	2.15	2.15	2.15	2.14	2.10
**Total number of included SNPs (%**^**a**^**)**	18,705 (50.5%)	17,505 (47.3%)	15,232 (41.1%)	13,186 (35.6%)	11,301 (30.5%)	9,476 (25.6%)	7,868 (21.2%)	6,256 (16.9%)	4,886 (13.2%)	2,991 (8.1%)
**Length of covered genome (Mb)**	249.7	230.6	196.8	168.2	142.3	117.6	96.0	75.3	57.1	32.2

^a^Number of included SNPs divided by total number of SNPs after quality control

Non-LD cluster SNPs were combined with pseudo-SNPs from haplotype blocks with different LD thresholds (Table 3). With an increase in ${r}^{2}$, the number of non-clustered SNPs decreased from 34,038 to 18,324, i.e., fewer SNP markers contributed to the haploblock construction, and more markers remained as individual SNP out of haploblocks. Notably, the proportion of pseudo-SNPs in the total variants decreased from 61 to 8% with an increase in the LD levels, and the total number of variants (i.e., the overall number of SNP and pseudo-SNP markers) decreased from 46,589 to 36,935.

**Table 3:** Number and proportion (%) of pseudo-SNPs and non-LD clustered SNPs combined for genomic prediction analyses, based on linkage disequilibrium (LD) level of ${\mathrm{r}}^{2}$

${\mathbf{r}}^{2}$	**Analysis**	**Pseudo-SNP (%)**	**SNP (%)**	**Total**
0.15	A_COM0.15_	28,265 (61%)	18,324 (39%)	46,589
0.20	A_COM0.20_	26,673 (58%)	19,527 (42%)	46,200
0.30	A_COM0.30_	22,971 (51%)	21,797 (49%)	44,768
0.40	A_COM0.40_	19,562 (45%)	23,843 (55%)	43,405
0.50	A_COM0.50_	16,384 (39%)	25,728 (61%)	42,112
0.60	A_COM0.60_	13,354 (33%)	27,553 (67%)	40,907
0.70	A_COM0.70_	10,663 (27%)	29,161 (73%)	39,824
0.80	A_COM0.80_	8,031 (21%)	30,773 (79%)	38,804
0.90	A_COM0.90_	5,816 (15%)	32,142 (85%)	37,958
1.00	A_COM1.00_	2,897 (8%)	34,038 (91%)	36,935

### Genomic relationship matrices

**Fig. 1:**
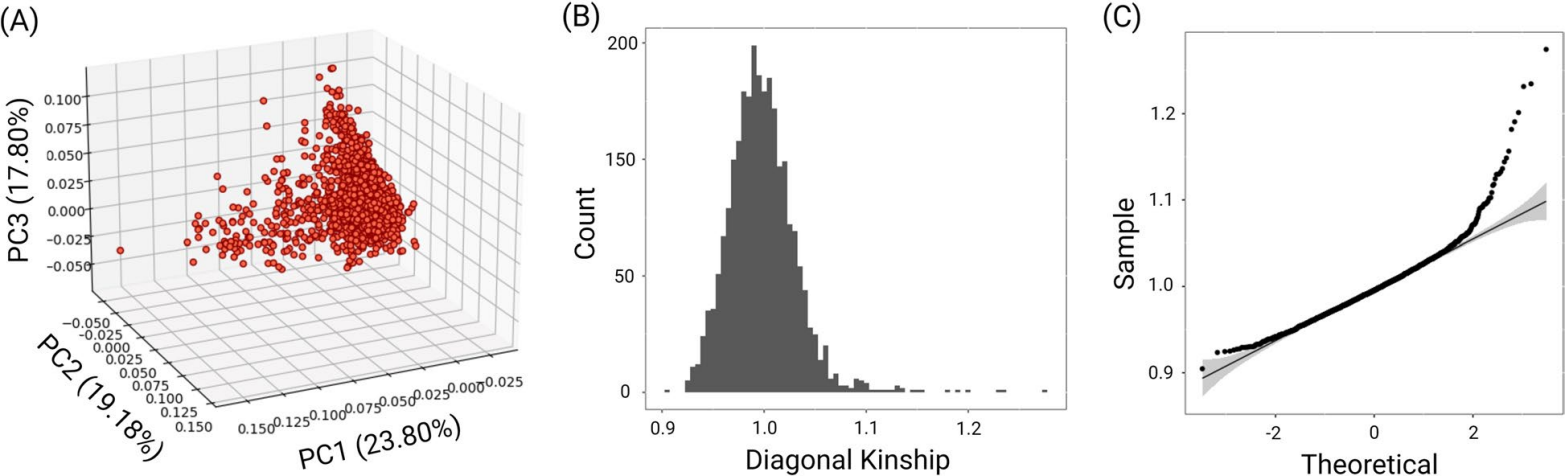
Plots of the principal components and the distribution of the genomic relationship matrix based on the genome-wide SNP markers. **A** Scatter plot of the first three principal components of the genomic dataset; no distinct clusters are observed in the studied dataset. **B** Histogram of the diagonal elements of the genomic relationship matrix. Despite the fact that a small portion of the population has high kinship values (> 1.1), no distinct peaks were observed for the studied dataset. (C) Quantile–quantile plot of the diagonal elements of the genomic relationship matrix. Overall, it is inferred that the genotypic dataset of Soay sheep was mainly generated from the animals with minimum family structure

The GRMs were constructed using the SNPs and pseudo-SNPs and based on the VanRaden method [11]. To investigate the presence of family structure in the studied population, the principal component analysis (PCA) of SNP markers was depicted (Fig. 1A). Furthermore, the distribution of the diagonal elements of the genomic relationship matrix based on individual SNP (G_SNP_) was plotted (Fig. 1B and Fig. 1C). The bar and QQ plots of G_SNP_ diagonal elements show that the distribution was close to normal. Moreover, no distinct cluster was observed in the PCA (Fig. 1A). These results confirm that there was minimal familial structure among the genotyped animals.

**Fig. 2:**
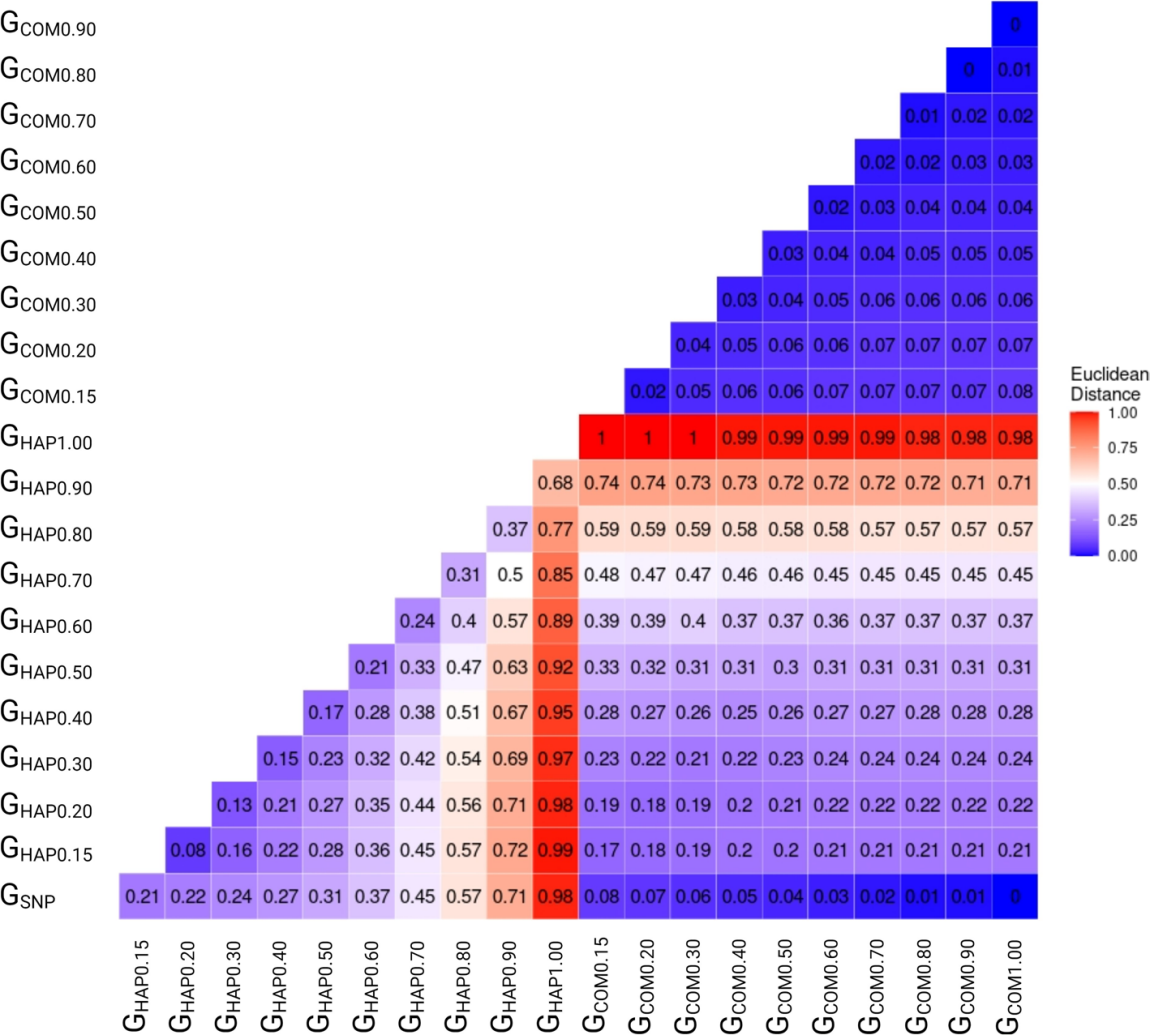
Heatmap of Euclidean distance between different genomic relationship matrices (GRM). Euclidean distance was used to compare a total of 21 GRMs, including G_SNP,_ which refers to the GRM defined based on SNPs as markers, and G_HAP0.15_, G_HAP0.20_, G_HAP0.30_, G_HAP0.40_, G_HAP0.50_, G_HAP0.60_, G_HAP0.70_, G_HAP0.80_, G_HAP0.90_, and G_HAP1.00_ defined based on haplotypes constructed by LD thresholds of 0.15, 0.20, 0.30, 0.40, 0.50, 0.60, 0.70, 0.80, 0.90, and 1.00 as markers, respectively. Values were scaled between 0 and 1

Euclidean distances between different pairs of GRMs are depicted in Fig. 2. Our results confirmed that GRMs constructed based on SNPs, haplotypes, or their combinations were different from each other. Regarding GRMs based on haplotypes, with stricter LD thresholds, more SNP markers were eliminated from the analyses; fewer of them contributed to the GRM construction. Therefore, with the increase in the LD threshold, higher distances were observed from G_SNP_. Concerning GRMs based on the combination of pseudo-SNPs and non-LD clustered SNPs, by increasing the LD threshold, more SNPs were removed from the haploblock construction; instead, they were used as individual SNP in the GRM construction. In contrast, with lower LD thresholds, more SNP markers would contribute to the haploblocks, and fewer non-LD clustered SNP markers were involved in the GRM construction. Consequently, with stricter LD thresholds, more similarity was observed between these GRMs and G_SNP_. Interestingly, the magnitude of differentiation from G_SNP_ was more apparent for GRMs based on the pseudo-SNPs than those observed for GRMs based on the combination of pseudo-SNPs and non-clustered SNPs at different levels of LD.

### Heritability estimates

**Fig. 3:**
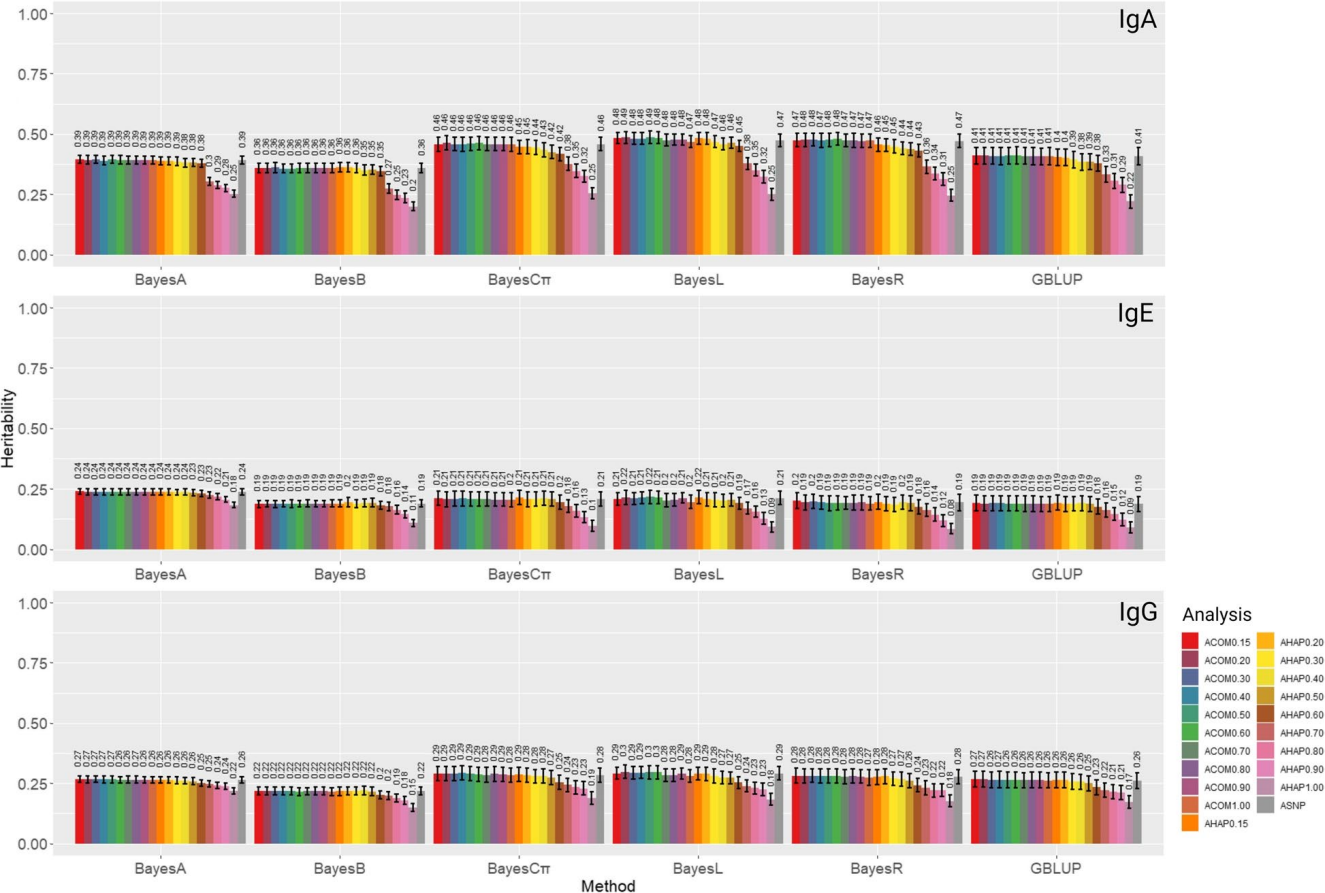
Heritability estimates for IgA, IgE, and IgG applying different methods and analyses. Heritability was computed as the ratio of the additive genetic variance to the phenotypic variance. The methods evaluated are GBLUB, BayesA, BayesB, BatesCπ, BayesLasso, and BayesR based on different analyses, including A_SNP_, A_COM0.15_, A_COM0.20_, A_COM0.30_, A_COM0.40_, A_COM0.50_, A_COM0.60_, A_COM0.70_, A_COM0.80_, A_COM0.90_, A_COM1.00_, A_HAP0.15_, A_HAP0.20_, A_HAP0.30_, A_HAP0.40_, A_HAP0.50_, A_HAP0.60_, A_HAP0.70_, A_HAP0.80_, A_HAP0.90_, and A_HAP1.00_. Definitions of the analyses are given in Tables 3 and 4

Heritability estimates for different scenarios are shown in Fig. 3. We observed higher ranges of heritability for IgA (0.20 to 0.49) compared to IgG (0.15 to 0.30) and IgE (0.08 to 0.24). The Bayesian methods resulted in higher heritability estimates for all traits than restricted maximum likelihood (REML). Irrespective of the applied methods, the heritability estimates were close when analyses were based on the combination of SNPs and pseudo-SNPs (A_COM0.15_-A_COM1.00_). However, for the analyses based on haplotypes (A_HAP0.15_-A_HAP1.00_), with more relaxed LD thresholds, from 1.00 to 0.15, the heritability estimates increased by 14–23%, 6–12%, and 3–11% for IgA, IgE, and IgG, respectively.

For each trait, the highest and lowest heritabilities obtained based on individual SNP, pseudo-SNP, and the combinations of pseudo-SNPs and non-LD clustered SNPs are listed in Supplementary Table 1. The highest heritability (0.49) was obtained for IgA when the BayesL method was applied to the A_COM0.20_ and A_COM0.50_. On the contrary, the lowest heritability (0.20) was observed when A_HAP1.00_ was applied to BayesB. Regarding IgE, the highest estimate (0.24) was obtained for BayesA based on A_SNP_, A_HAP0.15_, A_HAP0.20_, A_HAP0.30_, A_HAP0.40_, and based on all the applied combinations of SNPs and pseudo-SNPs. However, the lowest estimate of 0.08 was observed by BayesR with A_HAP1.00_. The highest heritability of IgG (0.30) was observed by BayesL based on A_COM0.20_, A_COM0.50_, and A_COM0.60_. In contrast, the lowest heritability of 0.15 was found by BayesB based on A_HAP1.00_.

### Genomic prediction accuracies

**Fig. 4:**
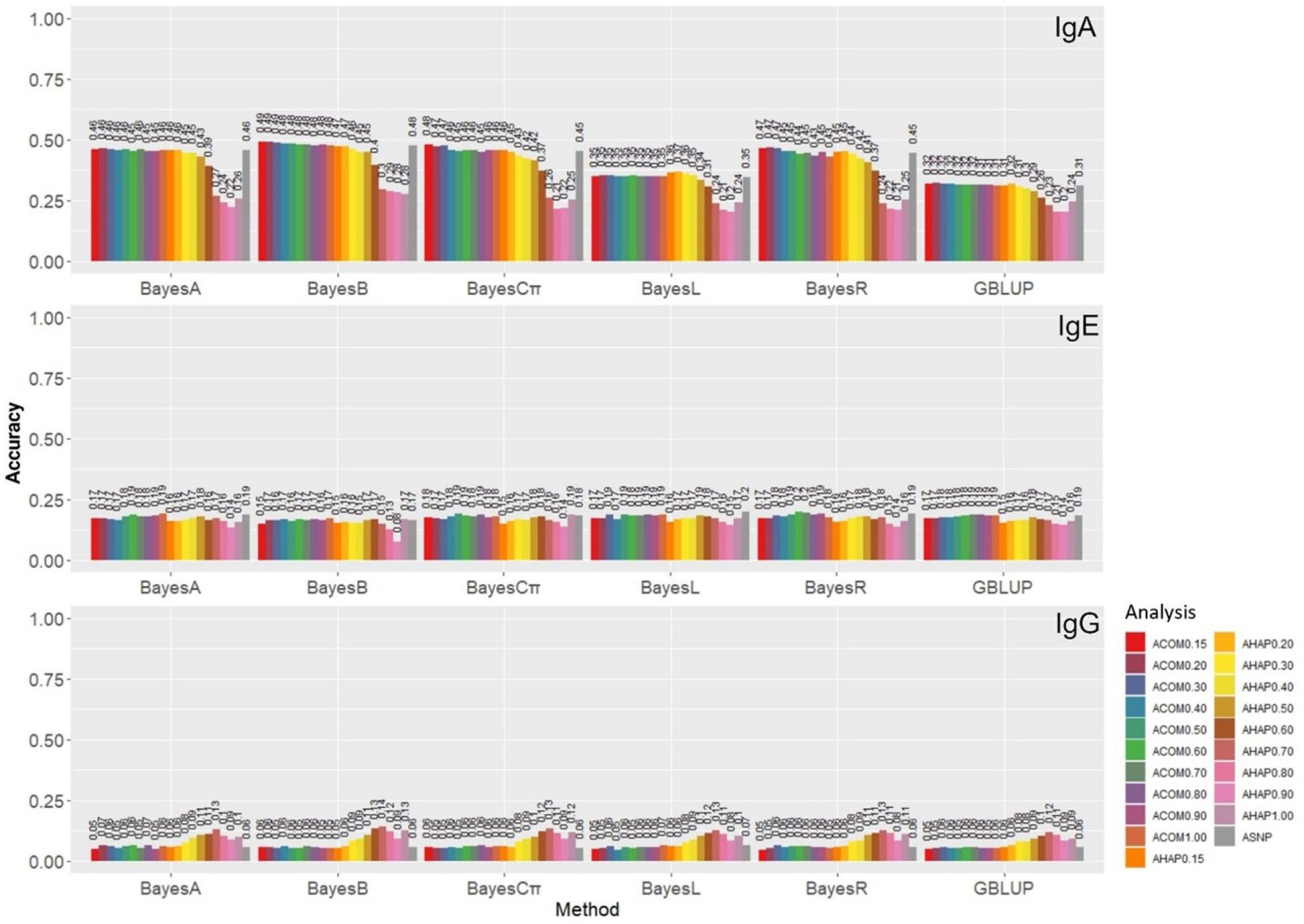
The estimates of genomic prediction accuracy of IgA, IgE, and IgG applying different methods and analyses. The genomic prediction accuracy was measured by the correlation between adjusted phenotypes (${\mathrm{y}}_{\mathrm{c}}$) and GEBV for the validation subset. Methods under evaluation were GBLUB, BayesA, BayesB, BatesCπ, BayesLasso, and BayesR based on different analyses, including A_SNP_, A_COM0.15_, A_COM0.20_, A_COM0.30_, A_COM0.40_, A_COM0.50_, A_COM0.60_, A_COM0.70_, A_COM0.80_, A_COM0.90_, A_COM1.00_, A_HAP0.15_, A_HAP0.20_, A_HAP0.30_, A_HAP0.40_, A_HAP0.50_, A_HAP0.60_, A_HAP0.70_, A_HAP0.80_, A_HAP0.90_, and A_HAP1.00_. Definitions of the analyses are given in Tables 3 and 4

The accuracies of GPs based on different methods and analyses are presented in Fig. 4. Higher ranges of accuracies were observed for IgA (0.20 to 0.49), followed by IgE (0.08 to 0.20), and IgG (0.05 to 0.14). Through comparing the accuracy of different marker sets in each applied method, we found up to 3% improvement in GP accuracy of IgA using the combination of the pseudo-SNPs with non-LD clustered SNPs. In contrast, for IgE, comparable accuracies to GBLUP using individual SNP were obtained by the combination of the pseudo-SNPs with non-LD clustered SNPs and haplotype-based GPs. For IgG, up to 8% gains in GP accuracy were observed in analyses based on the haplotypic pseudo-SNPs (Fig. 4).

Bayesian methods outperformed GBLUP in all three studied traits. By comparing the accuracy of different methods in each applied marker set, we found that BayesB outperformed all other methods for IgA. In most cases of IgE and IgG, Bayesian approaches obtained higher accuracy than GBLUP (Fig. 4). For each trait, the highest and lowest accuracies achieved based on individual SNP, pseudo-SNP, and the combinations of pseudo-SNPs and non-LD clustered SNPs were listed in Supplementary Table 2. Regarding IgA, the highest GP accuracy (0.49) was obtained with the BayesB method based on A_COM0.15_ or A_COM0.20_ compared to GBLUP using A_SNP_ (0.31). In contrast, the lowest accuracy (0.20) was estimated based on BayesL and GBLUP with A_HAP0.90_. Concerning IgE, the highest accuracy (0.20) was given by BayesL with A_SNP_ and BayesR with A_COM0.60_ or A_COM0.70_. On the contrary, the lowest accuracy (0.08) was obtained for BayesB using A_HAP0.90_. With regards to IgG, the highest accuracy (0.14) was estimated for BayesB based on A_HAP0.70_. Conversely, the weakest performance (0.04) was given by BayesL based on A_COM0.40_.

Regardless of the applied methods, a general trend was observed for IgA GPs based on haplotypic pseudo-SNPs and the combination of pseudo-SNPs and non-clustered SNPs, indicating lower accuracies with the increase in the LD threshold. Meanwhile, higher accuracies were obtained for IgG with higher LD threshold when the combinations of pseudo-SNPs and non-clustered SNPs were used; however, a slight reduction in the accuracies was observed with stringent LD levels (> 0.70).

In 13 out of the 18 model-trait combinations, i.e., all IgG scenarios along with five out of six IgA’s, at least one of the models based on haplotypes or the combination of haplotypes and non-clustered SNPs achieved a higher accuracy than the model fitting SNPs (Supplementary Table 3). Only in one scenario (BayesL for IgE), a higher accuracy was obtained by the model fitting individual SNPs. In four scenarios, comparable performances were observed between models fitting individual SNP and those using haplotypic information (Supplementray Table 3).

We revealed that the magnitude of differences between the highest and the lowest GP accuracies was higher for haplotype-based approaches than those based on the models fitting the combinations of pseudo-SNPs and SNP markers. These differences were 0.27, 0.11, and 0.09 for haplotype-based GPs of IgA, IgE, and IgG, respectively. In contrast, we obtained lower differences of 0.18, 0.05, and 0.02 for GPs of IgA, IgE, and IgG based on the combination of pseudo-SNPs and non-clustered SNP markers, respectively.

### Genomic prediction biases

**Fig. 5:**
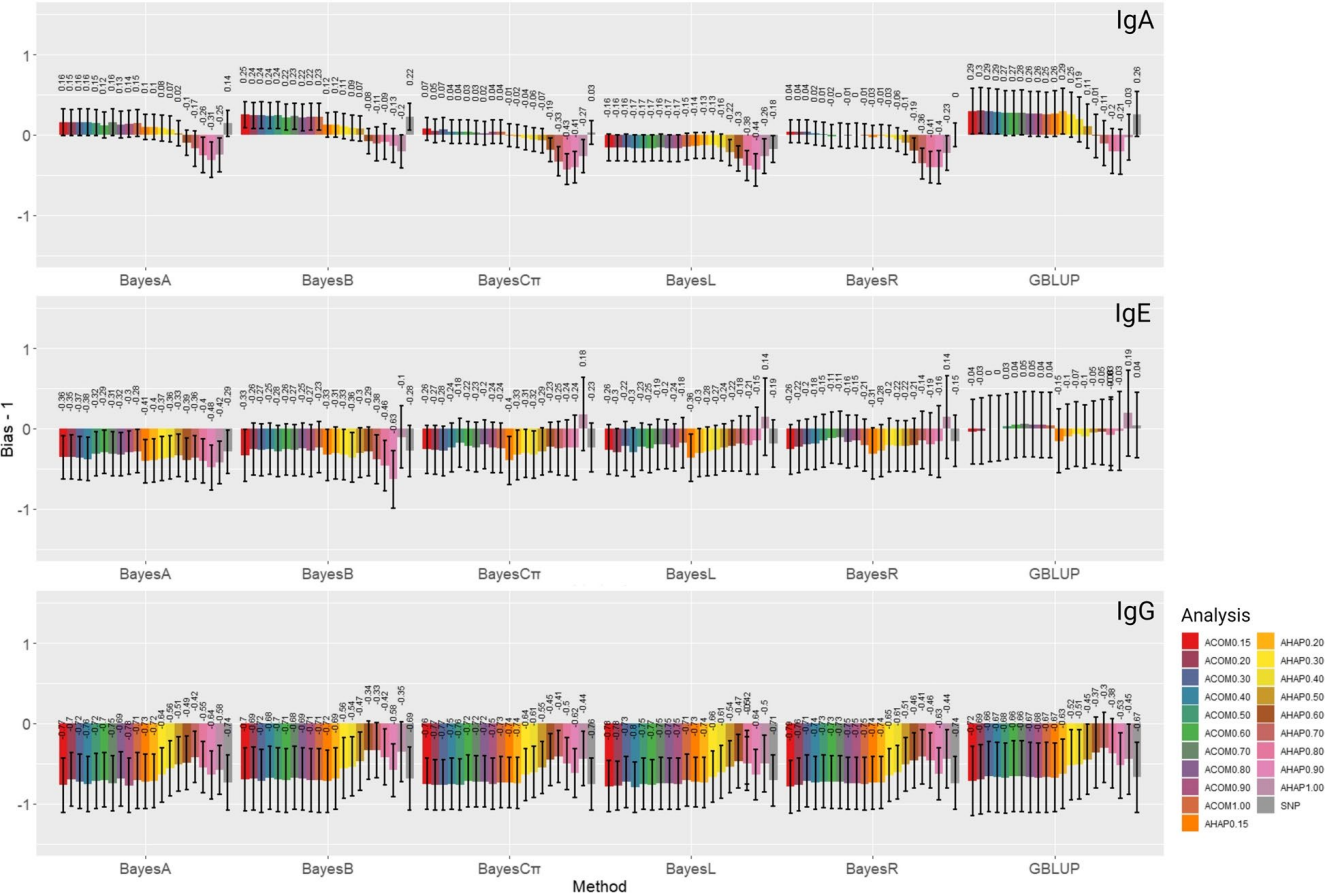
The bias estimates of genomic estimated breeding values (GEBV) of IgA, IgE, and IgG applying different methods and analyses. The genomic prediction bias was measured as the regression coefficients obtained by regressing the adjusted phenotypes (${\mathrm{y}}_{\mathrm{c}}$) upon the precited direct genomic values GEBV in the validation subset. Methods under evaluation were GBLUB, BayesA, BayesB, BatesCπ, BayesLasso, and BayesR based on different analyses, including A_SNP_, A_COM0.15_, A_COM0.20_, A_COM0.30_, A_COM0.40_, A_COM0.50_, A_COM0.60_, A_COM0.70_, A_COM0.80_, A_COM0.90_, A_COM1.00_, A_HAP0.15_, A_HAP0.20_, A_HAP0.30_, A_HAP0.40_, A_HAP0.50_, A_HAP0.60_, A_HAP0.70_, A_HAP0.80_, A_HAP0.90_, and A_HAP1.00_. Definitions of the analyses are given in Tables 3 and 4

The bias in Genomic Estimated Breeding Value (GEBV) predictions for all scenarios is presented in Fig. 5 as deviations from 1 (bias – 1). Considering all methods and analyses, the bias deviation values ranged from -0.44 to 0.30, -0.63 to 0.19, and -0.80 to -0.30 for IgA, IgE, and IgG, respectively. In most scenarios (17 out of 18 model-trait combinations), at least one of the models based on haplotypes or the combination of haplotypes and non-clustered SNPs achieved a lower bias than the model fitting SNPs individually (Supplementary Table 4). Only in one scenario a comparable bias was observed between models fitting individual SNPs and those using haplotypic information (Supplementary table 4).

For IgA, we observed unbiased GP by haplotype-based scenarios of BayesR using A_SNP_, A_COM0.70_, and A_COM0.90,_ which were comparable to the bias obtained by GBLUP using A_SNP_. In contrast, the most biased scenario was observed when BayesL was applied with A_HAP0.90_ (-0.44 ± 0.20). Regarding IgE, GBLUP based on A_COM0.30_ and A_COM0.40_ provided unbiased GEBV prediction. On the contrary, the highest level of bias was obtained by BayesB based on A_HAP0.90_ (-0.63 ± 0.36). Concerning IgG, the least biased haplotype-based GP was observed by GBLUP based on A_HAP0.70_ (-0.30 ± 0.70). However, the BayesL method using A_COM0.40_ showed the most biased GP (-0.80 ± 0.31). Haplotype-based GPs predicted less-biased GEBVs in most IgG scenarios with high LD thresholds compared with SNP-based models, whereas no improvement in bias was observed for other traits with an increase in LD level. Moreover, in most scenarios of IgE and all IgG scenarios, GEBV inflation (bias < 0) was observed.

## Discussion

GP has been widely used to enhance the genetic gain of complex traits in livestock and plant breeding and predict polygenic risk scores of particular human diseases [37–39]. Recently, more attention has been given to this tool in the evolutionary genetics topic to improve captive breeding strategies and understand the microevolution of breeding values [8, 9]. In this study, we applied a haplotypic GP approach on three helminth-specific immune response traits of IgA, IgE, and IgG against *T. circumcincta* in the unmanaged population of Soay sheep. Haplotype-based GP has achieved little to substantial improvements in prediction accuracies compared with SNP models in domesticated species [21, 22, 40], including sheep [41]. However, to our knowledge, the application of haplotype-based GP and assessment of haplotypic GP performance have not been reported in wild or unmanaged populations.

Haploblocks were constructed based on different LD thresholds (Table 2). The Big-LD method applied to our study constructs the LD blocks using the weights estimated based on the number of SNPs from all possible overlapping intervals [42]. We observed the construction of haploblocks with LD = 1, which is not very common in commercial populations of domesticated species, as markers in such a high LD are typically eliminated in the process of designing SNP panels. When setting low LD thresholds to construct the LD blocks, more intervals of linked SNPs are obtained, the number of blocks is increased, fewer SNPs are excluded, and a higher portion of the genome is covered by haploblocks (and vice versa). Consequently, a greater number of blocks are expected with lower LD thresholds, as observed when comparing the numbers of blocks across LD thresholds from 0.15 to 1.00 (Table 2). The average number of SNPs per block showed that most of the haploblocks were constructed by two SNPs; however, the proportion of two-SNP-blocks increased with stricter LD thresholds. The reason could be that we used genotypes obtained from a medium-density SNP chip (50 K) for haploblock construction. This could result in the less presence of haploblocks with > 2 SNP markers with high LD thresholds since one essential criterion by which SNP markers are selected for SNP chips in commercial species is the gaps between markers, more importantly, the distance between two adjacent SNPs. Moreover, genotype imputation based on higher-density reference panels can increase SNP density and, therefore, haplotype construction. However, higher-density SNP data was unavailable for the current study, and we suggest genotype imputation in future studies. The number of total variants increased for analyses based on haplotypic pseudo-SNPs and individual SNP with stricter LD thresholds (Table 3). The reason is that with higher LD, fewer individual SNP were blocked in haplotypes, and more SNP markers remained non-clustered.

We showed that with higher LD thresholds, GRMs constructed based on haplotypes are more differentiated than that based on individual SNP, whereas, for GRMs based on the combinations of pseudo-SNPs and non-clustered SNPs, the trend was reversed (Fig. 2). For GRMs based on haplotypes, with stricter LD thresholds, the relationship among individuals in the population is defined based on a shorter length of the genome and a lower number of SNPs; consequently, it could cause more differentiation among GRMs. However, for GRMs based on the combinations of pseudo-SNPs and non-clustered SNPs with higher LD thresholds, greater similarity was observed with G_SNP_ due to the lower number of blocked SNPs and more contribution of individual SNP to the GRM (Table 3).

### Heritability estimates

We estimated a wider range of heritabilities (0.08 to 0.49) for the antibody traits against *T. circumcincta* compared to previous studies on Soay lambs, where the estimated heritability of the antibody traits ranged from 0.21 to 0.39 [36, 43]. The differences among our estimated heritabilities and the previous studies could be due to the different methods, SNP/haplotype information, and models. For IgA and IgG, higher heritability estimates were achieved when a combination of haplotype information with non-clustered SNPs was used, and for IgE, haplotypes achieved an equal heritability to fitting individual SNPs (Supplementary Table 1). In all combinations of SNPs and non-clustered SNPs, the total number of variants were more than individual SNP or haplotypic pseudo-SNPs with different LD thresholds (Tables 3). Therefore, more variants were available to explain the phenotypic variances of IgA, IgE, and IgG. In parallel to our results, Won et al. [44] obtained higher heritability estimates from haplotypes than individual SNP for carcass traits in pigs. Estimated heritabilities among haplotype-based GPs tended to decrease as the LD threshold increased, the length of haplotypes shortened, and the number of haplotypes declined. With higher LD thresholds, a smaller number of SNP markers and shorter genomic length contributed to the haplotype block construction (Table 2). Therefore, fewer haplotypic pseudo-SNPs were available to explain the phenotypic variance, and a lower proportion of total variance could be captured, resulting in lower heritability estimates.

### Genomic prediction accuracy

Gains in the accuracy of IgG’s GPs were observed using haplotype-based pseudo-SNPs. Our results are in concordance with the previous studies, revealing that significant improvements in haplotype-based GPs could be gained when oligogenic traits or those affected by major genes are evaluated [22, 44, 45]. For instance, Won et al. [44] reported an increase of 4.6% in GEBV accuracy with haplotypic GP for eye muscle area in Korean cattle. Moreover, a 9.8% improvement in the accuracy of carcass weight GEBV was documented by incorporating haplotype information based on SNP markers from functionally related genomic regions [45]. Additionally, up to 22% gain in accuracy was observed using haplotypes from fixed length or LD blocking strategies in genomic evaluation of milk production traits in French dairy goats [22].

One explanation for the higher performance of haplotype-based GPs, particularly for IgG, could be that G_SNP_ is constructed based on marker alleles being IBS. As SNP chip markers typically represent old mutations, G_SNP_ mainly traces old relationships among distant relatives and may not precisely account for changes due to recent selection [19]. Meanwhile, haplotype blocks can provide more information on recent mutations and better show close relationships [25]. Another explanation is that haplotype blocks are multi-allelic; consequently, they can better capture the LD with multi-allelic QTLs than biallelic individual SNP [19]. Moreover, haplotype blocks are derived from common ancestors; thus, GRMs based on haplotypes can differentiate between IBD and IBS, while G_SNP_ lacks this ability [46]. Another advantage of haplotype information is that haplotypes include the local epistatic effects among QTLs located within the haplotype blocks [25]. We observed lower performances in GPs of IgA when haplotype-based pseudo-SNPs based on high LD thresholds were applied to analyses compared to individual SNP. This might be due to the reason that these haplotypes are not sufficient to capture the effects of all the important chromosomal regions controlling the trait.

In each applied method, gains in accuracy were observed for IgA when methods were applied based on the combination of pseudo-SNPs and non-clustered SNPs. Our results are in disagreement with the previous study in which the combinations of SNPs and non-clustered pseudo-SNPs were used for GP for the first time and showed no improvement in accuracy [23]. In their study, highly polygenic traits were simulated, and GP was performed based on the Single-step GBLUP method, while we used traits with different genetic architectures, and we conducted GP based on GBLUP and Bayesian methods. The difference in genetic architecture could result in capturing of a higher LD between the pseudo-SNPs and multiallelic QTLs when haplotypic pseudo-SNPs are added to individual SNP [47].

In all studied traits, Bayesian methods outperformed GBLUP. The genetic correlation between the studied traits was reported to be more than 69.5%, with the highest correlation of 82.4% between IgA and IgG [36]. Moreover, around 44% and 10% of additive genetic variance of IgA and IgG traits in Soay sheep were explained by three and two QTLs, with one overlapping genomic region on chromosome 20 [36]. The high genetic correlation between the studied traits, the overlapping QTLs between them, and the outperformance of Bayesian approaches for all the traits in the current study suggest that these traits are more likely to be less polygenic, with some similarities in their genetic architectures. In parallel to our results, several studies have shown that Bayesian approaches could yield higher GP accuracies than GBLUP for oligogenic traits [13, 48, 49].

Generally, the heritability of traits is positively associated with GP accuracy [50]. While we observed this trend in IgA and IgE, with higher LD thresholds, lower heritabilies and higher GP accuracies were obtained for IgG up to LD = 0.70. This could be due to the overlaps between the IgG major genes and the haplotypes constructed based on high LD thresholds of 0.60 and 0.70. We could not find any overlapped regions between the IgG QTLs identified by Sparks et al. [36] and the genomic positions of haplotypes in A_HAP0.70_; however, there might still be some unknown QTLs for this trait. Moreover, the obtained higher accuracies might be due to the better capture of LD between multiallelic QTLs, which could be missed in biallelic studies. For instance, the multiallelic polymorphism of the major histocompatibility complex region, which significantly contributes to antigen recognition and antibody production such as IgA, IgE, and IgG, has been well-documented in the Soay sheep population [51–53].

We achieved remarkably lower accuracies for IgG compared with IgA and IgE. While higher GP accuracies are more desirable, there are some explanations for why a trait may not evolve as expected, including: (a) there might be a genetic correlation between the trait of interest and fitness-related traits [54, 55], (b) considering the breeder’s equation ($R = {h}^{2}S$, where $R$ is the response to selection, ${h}^{2}$ is the narrow-sense heritability, and $S$ is the strength of selection; [56]), the fluctuations in environmental conditions covarying with the heritability of the trait [57], the strength of selection [58], or both [59], can affect the response to selection, (c) in particular situations, the trait has responded to the selection, but a change in environmental conditions caused the phenotypic trend to mask the underlying genetic trend, which is referred to as “cryptic microevolution” [35].

Considering the higher accuracy achieved in this study with haplotype-based GPs for IgG and with the combination of pseudo-SNPs and non-clustered SNPs for IgA, there is an opportunity to apply these models in evolutionary and conservation genetics to improve captive breeding strategies. Wild animals could be genotyped, and haplotype-based GP models could be used to select the best individuals for the traits of interest. Considering the rise in wildlife infectious diseases and the emergence of zoonotic infections in wild animal populations, haplotype-based GP models could be used to improve captive breeding and conservation strategies to select pathogen-resistant individuals. Meanwhile, GP models have already been successfully applied for better immune responses to pathogens in livestock species breeding programs, such as tuberculosis resistance in dairy cattle [60], resistance against *Piscirickettsia salmonis* in Atlantic salmon [61], and higher immune response for porcine reproductive and respiratory syndrome in pigs [62].

### Genomic prediction bias

The magnitude of the bias was lower for IgA among the three studied traits. The reason could be that bias in genomic evaluations was generally lower for the traits with higher heritability [63]. Less biased GPs were observed for some haplotypic scenarios in all traits (Supplementary Table 4). GP models using haplotypic pseudo-SNPs, which gained higher accuracy compared to those fitting individual SNP, predicted less-biased GEBVs for IgG. In contrast, the higher accuracy achieved by some combinations of pseuso-SNPs and non-clustered SNPs came with the cost of more-biased GEBVs for IgA. Karimi et al. [21] also reported a less biased GP based on haplotypic pseudo-SNPs for traits with moderate-to-high heritabilities in Holstein cattle. However, Feitosa et al. [24] observed a more biased prediction for beef fatty acid profile using the haplotype model compared with the SNP model. An explanation for the less biased genomic evaluation based on haplotypes in some haplotype-based scenarios could be that haploblocks account for local epistasis, i.e., the interaction between SNPs within haplotype block, which can reduce the bias of GPs [45, 64].

### Future studies

Several opportunities exist for additional assessments of haplotype-based GPs in wild populations. We investigated the application of haplotype-based GP on three traits with similar genetic architectures. Therefore, we suggest evaluating the performance of haplotype approaches on other traits with different genetic architectures. Also, the benefits of the haplotype-based methods need to be investigated with larger populations. We used the forward validation method to estimate GPs accuracy, as also used in other sheep GP studies [65]. With larger sample sizes, the accuracy of GPs could be evaluated based on alternative validation methods, such as random and k-means cross-validation. Furthermore, several other methods can be used for fitting haplotypes in GP analyses [25, 26, 47] and future studies could compare alternative methods. A well-known approach is using haplotype information in a multi-allelic mixed model treating each haplotype block as a locus and each haplotype within the haplotype block as an allele [66]. We assessed the efficacy of GP using haplotypes constructed based on different LD thresholds. Haplotypic pseudo-SNP can also be produced based on a fixed number of SNPs per haplotype block or a fixed block length.

## Conclusions

Haplotypic information could improve the accuracy of genomic evaluations for antibody production of IgG and IgA traits. The gains in accuracy were more remarkable for IgG in most scenarios applied pseudo-SNPs. The improvement in accuracy was more significant for IgA using some combinations of pseudo-SNPs with individual SNP, particularly when lower LD thresholds applied. However, the slightly higher accuracy in IgA comes with the cost of more bias compared to the SNPs. In all studied traits, Bayesian approaches outperformed GBLUP. Although genomic evaluations based on haplotypes require additional steps, achieved improvements in GEBVs accuracy for some traits could be advantageous. We anticipate that this method could be applied to evolutionary and conservation quantitative genetics research to improve captive breeding and conservation strategies and better understand unmanaged populations’ microevolution.

## Methods

### Study population

The St. Kilda archipelago (54o49′08o34’W) is located at 65 km west of the Outer Hebrides, Scotland, and consists of four islands: Hirta, Soay, Boreray, and Dun. Soay sheep are descendants of primitive European domestic sheep introduced to the island of Soay several millennia ago [67]. A population of unmanaged Soay sheep has inhabited the island of Hirta since 1932 [67]. The Hirta Soay sheep population is well-characterized by periods of growth followed by considerable declines due to cold winters, feed availability, and parasitic infections, leading to reduced body weight and increased mortality rates [68–70]. A longitudinal individual-based study on the Soay sheep population in the Village Bay area of Hirta began in 1985 [67]. Since that time, > 95% of the lambs have been captured during the lambing season in March–May to collect a variety of measures, including immunoglobulin (Ig) levels against *T. circumcincta* third larval stage [36, 71].

### Data preparation

A total of 2,061 IgA, IgE, and IgG records from 2,061 Soay sheep lambs against antigens of *T. circumcincta* were obtained from a publicly available dataset belonging to the study conducted by Sparks et al. [36]. In brief, antibody levels were measured as optical density (OD) values using direct ELISA tests on blood samples collected between 1990 and 2015. The procedure of capturing animals, sample collection, and ELISA methods were previously described in detail by Sparks et al. [36]. Samples belonging to the lambs within 50 days of birth were already removed from the dataset due to the potential presence of maternal antibodies [36]. Only animals with genotypic data were included in this study. Therefore, a total of 2,034 IgA, IgE, and IgG records belonging to 2,034 Soay lambs with an average ± SE, minimum and maximum age of 115.19 ± 0.17, 77, and 146 days remained for subsequent analyses.

A fixed-effects model was used to obtain the adjusted phenotypes for subsequent analyses. IgA, IgE, and IgG ODs were corrected for the systematic effects of animal age (in days), birth year, and sex. Two other “Plate ID” and “Run Date” effects were also present in the downloaded dataset. However, less than 5% of phenotypic variances of IgA, IgE, and IgG in lambs were explained by plate ID, and no variance was explained by Run Date in the Sparks et al. [36] study. Therefore, we did not use them in our analyses since they did not have remarkable effects. No information on other potentially significant effects was available in the dataset obtained. The fixed effect model was fitted using the AIREMLF90 package [72]. The residual effects were obtained and used as pseudo-phenotypes for the subsequent analyses.

Samples were already genotyped using the Illumina Ovine SNP50 BeadChip (Illumina; San Diego, CA, USA) by Sparks et al. [36]. While quality control was already conducted on the dataset by Sparks et al. [36], we reperformed quality control of 39,176 SNPs using PLINK 1.9 [73] on the lamb population (*N *= 2,034) with our criteria to ensure the quality of the data. Remaining markers with minor allele frequency (MAF) < 0.01, SNP calling rate < 0.90, extreme departure from Hardy–Weinberg equilibrium (*p*-value < 10^–6^), and SNPs located on non-autosomal chromosomes were removed. Moreover, samples with a genotype call rate of less than 90% were discarded from downstream analyses. A total of 37,031 SNPs from 2,034 lamb passed the quality control steps with the average genotype call rate > 99%. Then, the missing genotypes were imputed using the Beagle 5.2 software [74]. Subsequently, SNPs with MAF < 0.01 were filtered out. At the end, 37,029 SNPs from 2,034 lamb, all having the Ig records, remained for further analyses.

Principal component analysis was performed on SNP markers using the PLINK 1.9 software [73] to investigate the presence of family structure in the studied population. Furthermore, GRM was constructed by individual SNP (G_SNP_) using the AGHmatrix 2.0.4 R package [75] based on the VanRaden method [11]. Then, the distribution of the diagonal elements of the GRM was evaluated using bar and Q-Q plots to investigate the presence of significant family structures. All plots were created using the ggplot2 R package [76].

### Haploblock construction

The Big-LD method [42], which has been linked with higher accuracy in estimating the recombination hotspots than other existing methods, was used to construct the haplotype blocks. This method is based on interval graph modeling of LD bins which are clusters of strong pairwise LD SNPs, not necessarily physically consecutive [42]. As described by previous studies [23, 77], the gpart 1.13.0 package [78] in R [79] was used to implement the Big-LD method for haploblocks construction, using the default settings; however, the MAFcut was set to zero since the data was already passed this quality control test. Moreover, the CLQcut was set based on the common pairwise LD measure of ${r}^{2}$, and ten LD thresholds were considered, including 0.15, 0.20, 0.30, 0.40, 0.50, 0.60, 0.70, 0.80, 0.90, and 1.00. These LD thresholds were applied to capture different block structures from the biggest blocks with more SNPs in low LD (${r}^{2}$ = 0.15) to the smallest blocks with a lower number of SNPs in high LD (${r}^{2}$ = 1.00). Finally, the haplotype alleles were transformed to pseudo-SNPs, as described by Teissier et al. [22], using the GHap 2.0.0 R package [80]. Notably, many haploblocks can be multi-allelic, and several pseudo-SNPs can be created from the multiallelic haploblocks. The pseudo-SNPs were subjected to the same quality control criteria as the SNPs before their use for GP.

### Training and validation sets

We used the forward validation approach to evaluate the performance of the applied GPs models. The holdout approach has two main advantages over the common cross-validation approach, including: (i) in terms of breeding, holdout validation is generally preferred over cross-validation, as it provides a more realistic estimate of the accuracy of the model on new data. This is very important for breeding purposes, where the goal is to predict the genomic merit of future offspring based on the genotypes of their parents or individuals from previous generations. In contrast, in the cross-validation approach, animals from different generations can be denoted to the validation set, which is not realistic [65, 81, 82]; (ii) forward validation is computationally less intensive, as it requires training the model only once [13]. Forward validation approach has been widely used for evaluating GP models in different species, including sheep [83, 84], pigs [85], and cattle [86]. The validation set included lambs born in 2014 and 2015 (*N* = 186), comprising 10% of the total population. Lambs born before 2014 (*N* = 1,848) were classified as the training set and used to predict the GEBV of animals in the validation set. The size of the training and validation sets were comparable for all traits. Adjusted phenotypes calculated for animals born from 1995 to 2013 were used as pseudo-phenotype for the training, and those calculated for animals born in 2014 and 2015 were applied for validation.

### Genomic prediction of breeding values

Overall, six methods, including GBLUP, BayesA, BayesB, BayesCπ, BayesL, and BayesR were used in GP analyses. In each method, GP was computed based on three different analyses, including:

(i) analyses based on individual SNP (A_SNP_) fitted in the models;
(ii) analyses using haplotypes constructed based on different LD thresholds fitted as pseudo-SNPs in the GP models. Therefore, A_HAP0.15_, A_HAP0.20_, A_HAP0.30_, A_HAP0.40_, A_HAP0.50_, A_HAP0.60_, A_HAP0.70_, A_HAP0.80_, A_HAP0.90_, and A_HAP1.00_ refer to the analyses using haplotypes constructed by LD thresholds of 0.15, 0.20, 0.30, 0.40, 0.50, 0.60, 0.70, 0.80, 0.90, and 1.00 as pseudo-SNPs, respectively;
(iii) analyses using pseudo-SNPs in combination with non-LD clustered SNPs, located out of haploblocks, fitted in the model. After defining the haplotypic pseudo-SNPs based on the different LD thresholds, we combined them with individual SNP that were not blocked in haplotypes. Thus, A_COM0.15_, A_COM0.20_, A_COM0.30_, A_COM0.40_, A_COM0.50_, A_COM0.60_, A_COM0.70_, A_COM0.80_, A_COM0.90_, and A_COM1.00_ refer to the analyses in which the pseudo-SNPs with different LD thresholds were combined with non-LD clustered SNPs.

**GBLUP method:** The GBLUP model used was as follows:

$${\mathbf{y}}_{\mathbf{c}}=1\upmu +\mathbf{Z}\mathbf{g}+\mathbf{e},$$

where ${\mathbf{y}}_{\mathbf{c}}$ is the vector of adjusted phenotype in the reference population, $\upmu$ is the overall mean effect, $\mathbf{g}$ is the vector of additive genetic effects accounted for by all markers, i.e., SNPs in A_SNP_, pseudo-SNPs in A_HAP0.15_-A_HAP1.00_, or a combination of SNPs and pseudo-SNPs in A_COM0.15_-A_COM1.00_, and $\mathbf{e}$ is a vector of random residual. $\mathbf{Z}$ is the incidence matrix relating GEBV to adjusted phenotypes of individual animals. It was assumed that $\mathbf{g}\sim \mathrm{N}\left(0,\mathbf{G}{\upsigma }_{\mathrm{g}}^{2}\right)$ and $\mathbf{e}\sim \mathrm{N}\left(0,{\mathbf{I}\upsigma }_{\mathrm{e}}^{2}\right)$, where $\mathbf{G}$ is the GRM constructed based on only SNP markers, only haplotypes fitted as pseudo-SNPs, or a combination of pseudo-SNPs with non-clustered SNPs, **I** is an identity matrix, ${\upsigma }_{\mathrm{g}}^{2}$ is the additive genetic variance, and ${\upsigma }_{\mathrm{e}}^{2}$ is the residual variance. The GRM was constructed as follows [11]:

$$\mathbf{G}=\frac{\mathbf{Z}{\mathbf{Z}}^{\mathbf{^{\prime}}}}{2{\sum }_{\mathrm{j}=1}^{\mathrm{i}}{\mathrm{p}}_{\mathrm{j}}(1-{\mathrm{p}}_{\mathrm{j}})},$$

where, $\mathbf{Z}$ contains genotypes adjusted by the allele frequency and ${\mathrm{p}}_{\mathrm{j}}$ is the MAF of marker $\mathrm{j}$. Variance components were estimated using the Average Information Restricted Maximum Likelihood (AIREML) algorithm. This process and the prediction of GEBVs with GBLUP models were performed using the GVCBLUP software [87].

### Bayesian methods

Five Bayesian GP models were fitted, including BayesA, BayesB, BayesCπ, BayesL, and BayesR. For these methods, the general statistical model was:

$${\mathbf{y}}_{\mathbf{c}}=1\upmu +\sum_{\mathrm{j}=1}^{\mathrm{K}}{\mathbf{z}}_{\mathbf{j}}{\upbeta }_{\mathrm{j}}+\mathbf{e},$$

where, ${\mathbf{y}}_{\mathbf{c}}$ is the vector of adjusted phenotype in the reference population, $\upmu$ is the overall mean effect, $\mathrm{K}$ is the number of markers fitted, including SNPs in A_SNP_, pseudo-SNPs in A_HAP0.15_- A_HAP1.00_, or a combination of SNPs and pseudo-SNPs in A_COM0.15_-A_COM1.00_, ${\mathbf{z}}_{\mathbf{j}}$ is a vector denoting the genotypes of the animals for marker $\mathrm{j}$, ${\upbeta }_{\mathrm{j}}$ is the effect of marker $\mathrm{j}$, and $\mathbf{e}$ is a vector of random residuals. The vector of residuals $\mathbf{e}$ was assumed to be distributed as $\mathbf{e}\sim \mathrm{N}\left(0,{\mathbf{I}\upsigma }_{\mathrm{e}}^{2}\right)$, where ${\upsigma }_{\mathrm{e}}^{2}$ is the residual variance and **I** is an identity matrix. The hypothetical distribution of all markers’ effects in each Bayes method and the formula of the effect distribution are shown in Table 4.

**Table 4:** Assumption of effect size distribution of markers for the Bayesian methods used in this study

**Method**	**Joint distribution of SNP/ pseudo-SNP effect**	**SNP/pseudo-SNP effect distribution**^a^	**Variance distribution of the SNP/pseudo-SNP effects**	**Reference**
BayesA	t	${\upbeta }_{\mathrm{j}}$ $\sim$ N $(0,{\upsigma }_{\mathrm{g}}^{2})$	${\upsigma }_{\mathrm{g}}^{2} \sim {\upchi }^{-2}(\upnu ,\mathrm{ S})$	[1]
BayesB	point-t	${\upbeta }_{\mathrm{j}}$ $\sim 0.05\left(0,{\upsigma }_{\mathrm{g}}^{2}\right)+0.95{\updelta }_{0}$	${\upsigma }_{\mathrm{g}}^{2} \sim {\upchi }^{-2}(\upnu ,\mathrm{ S})$	[1]
BayesCπ	t mixture	${\upbeta }_{\mathrm{j}}$ $\sim$ $\left(1-\uppi \right)\mathrm{N}\left(0,{\upsigma }_{\mathrm{g}}^{2}\right)+\uppi {\updelta }_{0}$	${\upsigma }_{\mathrm{g}}^{2} \sim {\upchi }^{-2}(\upnu ,\mathrm{ S})$	[13]
BayesL	double exponential or Laplace	${\upbeta }_{\mathrm{j}}$ $\sim$ $\mathrm{N}\left(0,{\upsigma }_{\mathrm{g}}^{2}\right)$	${\upsigma }_{\mathrm{g}}^{2} \sim \mathrm{ Exp}(\frac{{\uplambda }^{2}}{2})$	[16]
BayesR	point-normal mixture	${\upbeta }_{\mathrm{j}}$ $\sim$ ${\uppi }_{1}{\updelta }_{0}+{\uppi }_{2}\mathrm{N}\left(0,{{10}^{-4}\upsigma }_{\mathrm{g}}^{2}\right)+{\uppi }_{3}\mathrm{N}\left(0,{{10}^{-3}\upsigma }_{\mathrm{g}}^{2}\right)+{\uppi }_{4}\mathrm{N}\left(0,{{10}^{-2}\upsigma }_{\mathrm{g}}^{2}\right)$	${\upsigma }_{\mathrm{g}}^{2} \sim {\upchi }^{-2}(\upnu ,\mathrm{ S})$	[88]

^a^where ${\upbeta }_{\mathrm{j}}$ is the effect of SNP/haplotype $\mathrm{j}$, ${\upsigma }_{\mathrm{g}}^{2}$ is the additive genetic variance, $\upnu$ and $\mathrm{S}$ are the degree freedom and scale parameter for inverse chi-square distribution, t represents student’s t-distribution, ${\updelta }_{0}$ is the effect size equals to zero, ${\uplambda }^{2}$ is the rate parameter which is assigned a gamma prior, $\uppi =({\uppi }_{1}+{\uppi }_{2}+{\uppi }_{3}+{\uppi }_{4})$ is the mixing proportions such that ${\sum }_{\mathrm{i}=1}^{4}{\uppi }_{\mathrm{i}}=1$

In all the Bayesian methods, the marker effects were estimated using a total of 100,000 Markov chain Monte Carlo (MCMC) iterations, with the first 20,000 discarded as burn in, and a thinning interval of 100. All the Bayesian methods were implemented using the hibayes R package [89]. We diagnosed convergence using a criterion of the accuracy of estimation of a quantile using the R package coda [90].

### Comparison of genomic relationship matrices

Overall, 21 GRMs were constructed for GPs, which could be classified into three categories:

(i) G_SNP_, which refers to the GRM defined based on SNPs as markers;
(ii) G_HAP0.15_, G_HAP0.20_, G_HAP0.30_, G_HAP0.40_, G_HAP0.50_, G_HAP0.60_, G_HAP0.70_, G_HAP0.80_, G_HAP0.90_, and G_HAP1.00_ defined based on haplotypes constructed by LD thresholds of 0.15, 0.20, 0.30, 0.40, 0.50, 0.60, 0.70, 0.80, 0.90, and 1.00 as markers, respectively;
(iii) G_COM0.15_, G_COM0.20_, G_COM0.30_, G_COM0.40_, G_COM0.50_, G_COM0.60_, G_COM0.70_, G_COM0.80_, G_COM0.90_, and G_COM1.00_ constructed using pseudo-SNPs with different LD thresholds combined with non-LD clustered SNPs.

As previously applied by Karimi et al. [21], to investigate the differences between matrices, pairwise Euclidean distance was calculated by $\mathrm{d}\left(\mathbf{C}, \mathbf{D}\right)=\sqrt{\sum_{\mathrm{i}}\sum_{\mathrm{j}}{({\mathrm{c}}_{\mathrm{ij}}-{\mathrm{d}}_{\mathrm{ij}})}^{2}}$, where ${\mathrm{c}}_{\mathrm{ij}}$ and ${\mathrm{d}}_{\mathrm{ij}}$ are elements of two comparing GRMs of **C** and **D**, respectively. Finally, the calculated values were scaled between 0 and 1.

### Heritability estimation

Variance components were estimated using the GVCBLUP software [87] and the hibayes package [89] for GBLUP and Bayesian approaches, respectively. The REML algorithm and MCMC method were applied to variance component estimation in GBLUP and Bayesian methods, respectively. In GBLUP, heritability was computed as ${\mathrm{h}}^{2}=\frac{{\upsigma }_{\mathrm{g}}^{2}}{{\upsigma }_{\mathrm{g}}^{2}+{\upsigma }_{\mathrm{e}}^{2}}$, where ${\upsigma }_{\mathrm{g}}^{2}$ and ${\upsigma }_{\mathrm{e}}^{2}$ are the additive genetic and residual variances, respectively. For the Bayesian methods, heritability was estimated by ${\mathrm{h}}^{2}=\frac{{\mathrm{V}}_{\mathrm{A}}}{{\mathrm{V}}_{\mathrm{A}}+{\upsigma }_{\mathrm{e}}^{2}}$. In this equation, ${\mathrm{V}}_{\mathrm{A}}$ is the total additive genetic variance which was estimated by ${\mathrm{V}}_{\mathrm{A}}=\uppi \times 2{\widehat{\upsigma }}_{\mathrm{SNP}}^{2}{\sum }_{\mathrm{j}=1}^{\mathrm{m}}{\mathrm{p}}_{\mathrm{j}}{\mathrm{q}}_{\mathrm{j}}$, where $\uppi$ is the proportion of the markers with non-zero effect, ${\widehat{\upsigma }}_{\mathrm{SNP}}^{2}$ is the marker variance, and ${\mathrm{p}}_{\mathrm{j}}$ and ${\mathrm{q}}_{\mathrm{j}}$ are allele frequencies of ${\mathrm{j}}^{\mathrm{th}}$ SNP or pseudo-SNP.

### Performance of genomic prediction of breeding values

The accuracy of the GEBV was obtained by dividing the Pearson correlation between adjusted phenotypes (${\mathrm{y}}_{\mathrm{c}}$) and GEBV for the validation subset. Bias was defined as the inflation or deflation of GEBV compared to adjusted phenotypes for the validation subset. The bias of the GEBV was calculated as the deviation from the unity of regression coefficient of adjusted phenotypes on GEBV for the validation subset [i.e., ${\mathrm{y}}_{\mathrm{c}}={\mathrm{b}}_{0}+{\mathrm{b}}_{1}\mathrm{GEBV}$ where ${\mathrm{y}}_{\mathrm{c}}$ is the adjusted phenotype in the validation set, GEBV corresponds to the predicted direct genomic values in the validation set, and ${\mathrm{b}}_{0}$ and ${\mathrm{b}}_{1}$ are the intercept and slope, respectively]. Therefore, the value of 0 indicates no bias in GEBV $\mathrm{estimates}$, while bias < 0 and > 0 show inflation and deflation, respectively [3, 91].

## Supplementary Information

**Additional file 1.**

## Data Availability

The datasets analyzed during the current study are available in the Dryad database: https://datadryad.org/stash/dataset/doi:10.5061/dryad.zgmsbcc6f and described in Sparks et al. [36]. All generated pseudo-SNPs, GEBVs, and scripts for the analyses are available at: https://osf.io/njm8v/

## Abbreviations

GP: Genomic prediction of breeding values
SNP: Single nucleotide polymorphism
LD: Linkage disequilibrium
REML: Restricted maximum likelihood
QTL: Quantitative trait loci
GBLUP: Genomic best linear unbiased prediction
BayesL: Bayesian Lasso
Ig: Immunoglobulin
GEBV: Genomic estimated breeding values
IBD: Identical-by-descent
GRM: Genomic relationship matrix
OD: Optical density
SE: Standard error
PCA: Principal component analysis
MAF: Minor allele frequency
AIREML: Average information restricted maximum Llremlikelihood
MCMC: Markov chain Monte Carlo
