# Correction: Expanding the application of haplotype-based genomic predictions to the wild: a case of antibody response against *Teladorsagia circumcincta* in Soay sheep

**DOI:** 10.1186/s12864-024-10797-y

**Published:** 2024-09-26

**Authors:** Seyed Milad Vahedi, Siavash Salek Ardestani, Luiz F. Brito, Karim Karimi, Kian Pahlavan Afshari, Mohammad Hossein Banabazi

**Affiliations:** 1 Department of Animal Science and Aquaculture, Dalhousie University, Truro, NS B2N5E3 Canada; 2 Department of Animal Science, University of Zanjan, Zanjan, 4537138791 Iran; 3 Department of Animal Sciences, Purdue University, West Lafayette, IN 47907 USA; 4 Molecular Diagnostics Program, London Health Sciences Centre, Verspeeten Clinical Genome Centre, London, ON N6A 5W9 Canada; 5 Department of Animal Sciences, Islamic Azad University, Varamin Varamin-Pishva Branch 3381774895, Tehran, Iran; 6 Department of Animal Breeding and Genetics (HGEN), Centre for Veterinary Medicine and Animal Science (VHC), Swedish University of Agricultural Sciences (SLU), Uppsala, 75007 Sweden; 7 Department of Biotechnology, Agricultural Research, Education & Extension Organization (AREEO), Animal Science Research Institute of IRAN (ASRI), Karaj, 3146618361 Iran

**Correction: BMC Genomics 24**,** 335 (2023)**

10.1186/s12864-023-09407-0

Following publication of the original article it was reported that there was an error in Fig. 4 and in the ‘Future studies’ subsection.

In Fig. 4 the legend title was ‘SH’ and has been corrected to ‘Analysis’. The correct Fig. 4 is provided in this Correction.

**Fig. 4:**
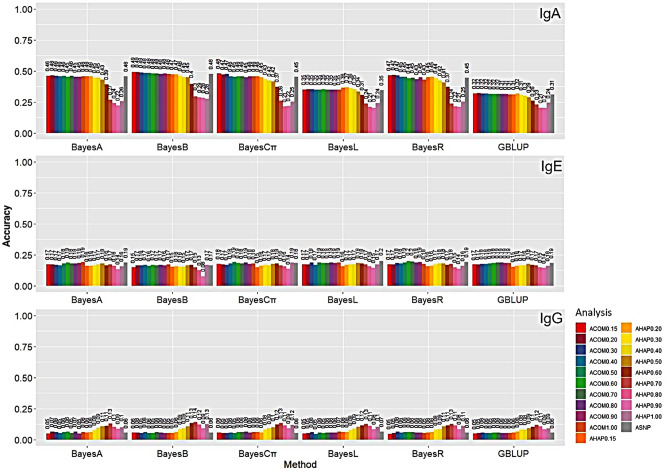
The estimates of genomic prediction accuracy of IgA, IgE, and IgG applying diferent methods and analyses. The genomic prediction accuracy was measured by the correlation between adjusted phenotypes (yc) and GEBV for the validation subset. Methods under evaluation were GBLUB, BayesA, BayesB, BatesCπ, BayesLasso, and BayesR based on diferent analyses, including ASNP, ACOM0.15, ACOM0.20, ACOM0.30, ACOM0.40, ACOM0.50, ACOM0.60, ACOM0.70, ACOM0.80, ACOM0.90, ACOM1.00, AHAP0.15, AHAP0.20, AHAP0.30, AHAP0.40, AHAP0.50, AHAP0.60, AHAP0.70, AHAP0.80, AHAP0.90, and AHAP1.00. Defnitions of the analyses are given in Tables 3 and 4

In the ‘Future studies’ subsection the following sentence had the word ‘references’ in place of missing references: “Furthermore, several other methods can be used for fitting haplotypes in GP analyses (references), and future studies could compare alternative methods.”

The correct sentence including the missing references is: “Furthermore, several other methods can be used for fitting haplotypes in GP analyses [25, 26, 47], and future studies could compare alternative methods.”

The original article has been updated.

