# Supplementary material for: Expanding the application of haplotype-based genomic predictions to the wild: A case of antibody response against Teladorsagia circumcincta in Soay sheep
Source: BMC Genomics. 2023 Jun 17;24:335. doi: 10.1186/s12864-023-09407-0 (PMC10276919; doi:10.1186/s12864-023-09407-0)
Supplement: Supplementary file 1 — Additional file 1. [file 12864_2023_9407_MOESM1_ESM.docx]

**Supplementary Tables**

**Table S1. Highest and lowest heritabilities (**$\mathbf{h}^{\mathbf{2}}$**) for IgA, IgE, and IgG obtained based on individual SNP, haplotypic pseudo-SNP from blocks with different linkage disequilibrium (LD) thresholds (0.15, 0.2, 0.3, 0.4, 0.5, 0.6, 0.7, 0.8, 0.9 and 1.00), and the combinations of pseudo-SNPs and non-LD clustered SNPs^a^. Values in bold^b^ indicate the highest estimated heritability for the trait among all scenarios.**

| Trait | Scenario | SNP | | Pseudo-SNP | | | Combined^a^ | | |
| --- | --- | --- | --- | --- | --- | --- | --- | --- | --- |
|  |  | **Method** | $\mathbf{h}^{\mathbf{2}}$ | **Method** | $\mathbf{h}^{\mathbf{2}}$ | **LD** | **Method** | $\mathbf{h}^{\mathbf{2}}$ | **LD** |
| IgA | Highest | BayesL  BayesR | 0.47±0.03  0.47±0.03 | BayesL  BayesL | 0.48±0.02  0.48±0.03 | 0.15  0.20 | **BayesL**  **BayesL** | **0.49±0.03^b^**  **0.49±0.02** | **0.50**  **0.20** |
|  | Lowest | BayesB | 0.36±0.02 | BayesB | 0.20±0.02 | 1.00 | BayesB | 0.36±0.02 | 0.15-1.00 |
| IgE | Highest | **BayesA** | **0.24±0.01** | **BayesA** | **0.24±0.01** | **0.15-0.40** | **BayesA** | **0.24±0.01** | **0.15-1.00** |
|  | Lowest | GBLUP  BayesB | 0.19±0.01  0.19±0.03 | BayesR | 0.08±0.02 | 1.00 | BayesB  BayesB  BayesR  GBLUP | 0.19±0.01  0.19±0.02  0.19±0.03  0.19±0.03 | 0.20-0.40 & 0.50-1.00  0.15 & 0.50  0.20 & 0.40-1.00  0.15-0.90 |
| IgG | Highest | BayesL | 0.29±0.03 | BayesL  BayesCπ | 0.29±0.03  0.29±0.03 | 0.15 & 0.20  0.15 | **BayesL** | **0.30±0.03** | **0.20 & 0.50-0.60** |
|  | Lowest | BayesB | 0.22±0.02 | BayesB | 0.15±0.02 | 1.00 | BayesB | 0.22±0.02 | 0.15-1.00 |

**Table S2. Highest and lowest accuracies of genomic predictions for IgA, IgE, and IgG achieved based on individual SNP, haplotypic pseudo-SNP from blocks with different linkage disequilibrium (LD) thresholds (0.15, 0.2, 0.3, 0.4, 0.5, 0.6, 0.7, 0.8, 0.9 and 1.00), and the combinations of pseudo-SNPs and non-LD clustered SNPs^a^. Values in bold^b^ indicate the highest accuracy achieved for the trait in the applied method.**

| **Trait** | **Scenario** | **SNP** | | **Pseudo-SNP** | | | **Combined^a^** | | |
| --- | --- | --- | --- | --- | --- | --- | --- | --- | --- |
|  |  | **Method** | **Accuracy** | **Method** | **Accuracy** | **LD** | **Method** | **Accuracy** | **LD** |
| IgA | Highest | BayesB | 0.48 | BayesB | 0.47 | 0.15 & 0.20 | **BayesB** | **0.49^b^** | **0.15 & 0.20** |
|  | Lowest | GBLUP | 0.31 | BayesL  GBLUP | 0.20  0.20 | 0.90  0.90 | GBLUP | 0.31 | 0.80-1.00 |
| IgE | Highest | **BayesL** | **0.20** | BayesCπ  BayesL | 0.19  0.19 | 1.00  0.50 | **BayesR** | **0.20** | **0.60 &0.70** |
|  | Lowest | BayesB | 0.17 | BayesB | 0.08 | 0.90 | BayesB | 0.15 | 0.15 |
| IgG | Highest | BayesL | 0.07 | **BayesB** | **0.14** | **0.70** | BayesA | 0.07 | 0.20 & 0.80 & 1.00 |
|  | Lowest | BayesCπ | 0.06 | BayesB | 0.05 | 0.15 | BayesL | 0.04 | 0.40 |

**Table S3. The highest accuracy achieved by haplotypic pseudo-SNP^a^ from blocks with different linkage disequilibrium (LD) thresholds and the combinations of pseudo-SNPs and non-LD clustered SNPs^b^, and the one obtained by SNPs for each applied method and trait. Values in bold^c^ indicate the highest accuracy achieved for the trait in the applied method.**

| **Trait** | **BayesA** | | | **BayesB** | | | **BayesCπ** | | | **BayesL** | | | **BayesR** | | | **GBLUP** | | |
| --- | --- | --- | --- | --- | --- | --- | --- | --- | --- | --- | --- | --- | --- | --- | --- | --- | --- | --- |
|  | **SNP** | **Pseudo^a^** | **Com^b^** | **SNP** | **Pseudo** | **Com** | **SNP** | **Pseudo** | **Com** | **SNP** | **Pseudo** | **Com** | **SNP** | **Pseudo** | **Com** | **SNP** | **Pseudo** | **Com** |
| IgA | **0.46^c^** | **0.46** | **0.46** | 0.48 | 0.47 | **0.49** | 0.45 | 0.46 | **0.48** | 0.35 | **0.37** | 0.35 | 0.45 | 0.45 | **0.47** | 0.31 | **0.32** | **0.32** |
| IgE | **0.19** | 0.18 | **0.19** | **0.17** | **0.17** | **0.17** | 0.18 | **0.19** | **0.19** | **0.20** | 0.17 | 0.19 | 0.19 | 0.18 | **0.20** | **0.19** | 0.18 | **0.19** |
| IgG | 0.06 | **0.13** | 0.07 | 0.06 | **0.14** | 0.06 | 0.06 | **0.13** | 0.06 | 0.07 | **0.13** | 0.07 | 0.06 | **0.13** | 0.06 | 0.06 | **0.12** | 0.06 |

**Table S4. The lowest 1-bias (closest to zero) achieved by haplotypic pseudo-SNP^a^ from blocks with different linkage disequilibrium (LD) thresholds and the combinations of pseudo-SNPs and non-LD clustered SNPs^b^, and the one obtained by SNPs for each applied method and trait. Values in bold^c^ indicate the lowest bias achieved for the trait in the applied method.**

| **Trait** | **BayesA** | | | **BayesB** | | | **BayesCπ** | | | **BayesL** | | | **BayesR** | | | **GBLUP** | | |
| --- | --- | --- | --- | --- | --- | --- | --- | --- | --- | --- | --- | --- | --- | --- | --- | --- | --- | --- |
|  | **SNP** | **Pseudo^a^** | **Com^b^** | **SNP** | **Pseudo** | **Com** | **SNP** | **Pseudo** | **Com** | **SNP** | **Pseudo** | **Com** | **SNP** | **Pseudo** | **Com** | **SNP** | **Pseudo** | **Com** |
| IgA | 0.14 | **0.02^c^** | 0.12 | 0.22 | 0.22 | **0.07** | 0.03 | **-0.01** | 0.02 | -0.18 | **-0.13** | -0.15 | **0** | -0.01 | **0** | 0.26 | **-0.01** | 0.25 |
| IgE | -0.29 | -0.33 | **-0.28** | -0.28 | **-0.10** | -0.23 | -0.23 | **0.18** | **-0.18** | -0.19 | **0.14** | -0.18 | -0.15 | ±0.14 | **-0.11** | 0.04 | -0.03 | **0** |
| IgG | -0.74 | **-0.42** | -0.69 | -0.69 | **-0.33** | -0.68 | -0.76 | **-0.41** | -0.72 | -0.71 | **-0.42** | -0.71 | -0.74 | **-0.41** | -0.71 | -0.67 | **-0.30** | -0.66 |
